# Triple Antibiotic Paste: A Suitable Medicament for Intracanal Disinfection

**DOI:** 10.7759/cureus.29186

**Published:** 2022-09-15

**Authors:** Krutika Malu, Monika Khubchandani

**Affiliations:** 1 Pediatric Dentistry, Sharad Pawar Dental College, Datta Meghe Institute of Medical Sciences, Wardha, IND

**Keywords:** dental treatment, intracanal disinfectant, triple antibiotic paste, pulp regeneration, endodontic treatment, apexification, antibiotic

## Abstract

With increasing cases of odontogenic infections, advancements in the treatment modality gain utmost importance. Complexity in the anatomy of the root canal necessitates the selection of the correct medicament and disinfectant. Furthermore, exacerbation of the problem results due to improper cleaning and disinfection of the root canal space. In such cases, manual preparation and irrigation alone will be of no help. The treatment outcome mostly depends upon the correct selection and application of the proper intracanal disinfectant along with the proper choice of medicament. One such intracanal disinfectant is triple antibiotic paste (TAP), a mix of three antibiotics. It’s the combined effect of the three drugs mixed in the paste that makes the mix a potent antimicrobial agent effective against microbes. This review aims to evaluate the properties of TAP, its composition, its various application, and its property to help maintain the vitality of the diseased pulp. This review also talks about its drawbacks and its application in primary teeth.

## Introduction and background

Endodontic treatments fall under the criteria of critical importance and advanced dental procedures because they ensure appropriate functioning and preservation of the tooth and integrity of the dental arch in the oral cavity [1]. Various approaches have been offered, ranging from old preparatory techniques to modern ones. Paediatric endodontics is concerned with pulp therapy for deciduous teeth. In case of irreversible damage to the pulp of the deciduous tooth, endodontic therapy is performed to keep the tooth in a functional state until its scheduled exfoliation period. Root canal medicaments, particularly antibiotics, have steadily revealed their crucial importance and key role in achieving positive outcomes as an adjunct to clinical therapy.

Odontogenic infections refer to infections arising from the structures of the teeth. These infections, like any other, involve numerous organisms that demand the action of multiple antibiotics to combat the species responsible for the lesion [2]. Thus, antibiotics today have become a non-separable part of dental treatment. Antibiotics with different formulas are used to treat different diseases and for prophylactic reasons as well [3]. Effective decontamination is a must to treat odontogenic lesions related to endodontic causes successfully. Although endodontic instruments and irritants are the primary means of decontaminating and disinfection, certain conditions demand the use of the medicaments in the canal region, considering the predominance of microorganisms. The importance of intracanal medications cannot be overstated, particularly in the event of recalcitrant lesions. Intracanal medicaments are temporarily placed materials for the purpose of creating a sterile environment in the root canals, which are populated with pathogenic microbes [3,4]. In chronic, long-standing cases of infection, the complexity of root canal anatomy shelters the endo-pathogens from the effect of irrigation and instrumentation. A single antibiotic is insufficient to eliminate pathogenic flora; hence a combination of drugs popularly known as ''triple antibiotic paste'' (TAP) is recommended. This mix is potently active against a wide range of bacteria, be it obligatory or facultative, gram-positive or gram-negative, allowing the site to heal. It aids in disinfecting and sterilising the root canal system by making the area free of microbial colonization. It allows the entry and growth of new tissue in the radicular area in regenerative therapy in the case of young, immature teeth. TAP can also assist in the development of a discipline that will allow for the successful application of other desired and necessary therapies. Finally, owing to its outstanding property of disinfection and regeneration, TAP has excellent applications as an antibacterial intracanal disinfectant [5,6].

The ideal requirements of intracanal medicaments include: (i) the drug should not be irritating in nature, (ii) it should not cause staining, (iii) it should be highly effective in order to be used widely and referred to as biocompatible, and (iv) it should be able to effectively create positive outcomes over an extended period of time and be excellent at repairing the injured peri-radicular tissue.

TAP is extremely effective in the treatment of weeping canals. Additionally, it can also be applied to neutralize tissue debris, destroy any lingering microorganisms in the canal, and prevent dressing leaks.

## Review

TAP

TAP is an "intra-canal medicament", which happens to have three antibiotics combined in it, namely metronidazole, ciprofloxacin, and minocycline, in a definite proportion of 1:1:1 to produce effective results. Considering that a single antibiotic is not potent enough to remove all polymicrobial flora, TAP is employed to obtain the best results and thoroughly disinfect the area. Being a combination of three antibiotics, TAP has effective antibacterial effects in endodontic regenerative operation. With the help of its cleansing and sterilizing action, the canal paves the way for the cells carrying the potential to regenerate. The combined action of the three medications in a single mix is extremely effective as the chances of microbial resistance are reduced to a significant percentage. TAP is not only an effective antimicrobial agent but also possesses a good regeneration-inducing potential. It enables the proliferation of the stem cells in the apical region, resulting in the obtainment of the apical barrier [7].

History

Grossman utilized a poly-antibiotic formula known as PBSC, a combination of "penicillin, bacitracin, streptomycin, and caprylate sodium" in a silicon medium in a paste form, for the first time in endodontics in 1951 [7,8]. Penicillin was used to treat gram-positive bacteria, bacitracin was used to treat penicillin-resistant bacteria, streptomycin was used to treat gram-negative bacteria, and sodium caprylate was used to treat yeasts. Although the clinical evaluation of PBSC revealed therapeutic results, the formula was ineffective against anaerobic microbes, which play a key role in endodontic disorders. As a result, in 1975, the United States Food and Drug Administration banned PBSC for endodontic use, citing the risk of sensitization and penicillin allergy [8]. In 2006, the American Association of Endodontics released an article on the use of antibiotics to control root canal bacteria, which appear to be important in the aetiology and progression of pulpitis [8]. TAP is an antibiotic combination that has been explicitly designed for the rejuvenation process. Hoshino and colleagues introduced it after researching its effectiveness in removing the microbes from the root canal [9]. This mix, being a potent anti-microbial agent, has a wide range of applications in the field of endodontics, and is used in the treatment of necrotic pulp in open apex teeth [8,9].

Concentration for usage 

TAP is prepared in two ways: (i) Mixing ciprofloxacin, metronidazole, and minocycline in a proportion of 1:1:1, that is ciprofloxacin 33%, metronidazole 33%, and minocycline 34%, with macrogol and propylene glycol paste concentrated at 0.1-1.0 mg/ml; (ii) Mixing ciprofloxacin, metronidazole, and minocycline in a proportion of 1:3:3 [10-12].

Objective behind combining the antibiotics

Conventional antibiotics, when used individually, are unable to generate a "bacteria-free zone" in the canal due to the involvement of diverse microbiota in tooth infection. Additionally, antibiotic medication may also eliminate the native bacterial flora in the canal, resulting in a more aggressive infection. As a result, to avoid microbial resistance, all endodontic illnesses must be treated with a mix of antibiotics [13-15].

Components of TAP

Minocycline

Minocycline belongs to a class of antibiotics known as "broad-spectrum antibiotics", which is effective against an extensive range of bacteria. Being bacteriostatic, it possesses the advantage of not releasing antigenic products in the infected region. Apart from being an excellent antibacterial substance, it also has a regeneration-inducing property as it inhibits the action of clastic cells and stops the activity of collagenases. Doing this permits the growth of natural healthy cells and helps in the regenerative procedure [16,17].

Metronidazole

Metronidazole is a nitroimidazole chemical with a wide range of anti-anaerobic and anti-protozoal activity. Because of its potent activity against a wide range of bacteria and anaerobic cocci, it has been widely used in both local and systemic forms. It kills the bacteria by perforating and entering their membranes and attaching itself to their DNA, causing the helical structure to be disrupted and the cell to die quickly. Metronidazole prevents the development of all obligatory anaerobes examined and outperforms calcium hydroxide against two of the strains [18-21].

Ciprofloxacin

Ciprofloxacin belongs to a class of second-generation fluoroquinolone antibiotics used to treat bacterial infections that cause stomach pain, diarrhoea, and urinary tract infections. Ciprofloxacin and other fluoroquinolones are used for various purposes, in both oral and intravenous forms, because of their high tissue penetration [22].

Applications of TAP

TAP, a potent antimicrobial agent, is used in several forms and for several purposes. It is used to preserve the vitality of the pulp by trying to "regenerate and revascularize" the diseased pulp. TAP is not only a potent disinfecting agent but also plays a vital role in regeneration and revascularization [23]. The result of increased root length is observed more commonly with the use of TAP and calcium hydroxide than with the use of non-surgical root canal treatment and mineral trioxide aggregate (MTA) apexification. Revascularization is the process of inducing bleeding in the root canal, which in turn helps carry stem cells from the periapical region, and helps in the root lengthening procedure. As it has an antimicrobial effect when used in the root canal, it shows a dual effect - first by acting against *Enterococcus faecalis*, which is the most prevailing microbe in the root canal region, and next by bringing in the stem cells, followed by their proliferation, which helps expand the root length [24,25]. An important application of TAP is as an intracanal medicament in the cases of periapical lesions, external inflammatory root resorption, root fracture, and treatment flareups, which is a common complication of endodontic treatment. Flare-up is a condition characterized by the onset of severe pain and swelling after an endodontic therapy appointment as a result of an acute exacerbation of an existing condition demanding an unplanned treatment appointment [26].

Use of TAP in the treatment of primary teeth

TAP and Vitapex® (J. Morita Corporation, Kyoto City, Japan) had substantial results in root canal treatment of infected primary teeth, according to Nakornchai et al. [27]. The success rate for TAP is 96% [27]. At 24-27 months after surgery, researchers looked at the clinical and radiographic success rates of TAP in non-instrumentation endodontic treatment of primary mandibular molars; despite a low success rate based on a two-year radiographic study, the findings demonstrated that this approach had a high success rate [28]. Takushige et al. studied TAP's effect on the clinical outcome of lesion sterilization and tissue repair treatment in primary teeth with peri-radicular lesions. Clinical symptoms such as sinus tracts, gingival swelling, dull soreness, and other symptoms disappeared after treatment. The teeth that were effectively treated appeared and normally erupted on radiographs [29,30].

Effect of TAP on tooth structure

Effect on Dentin

Studies have reported that TAP has a demineralizing effect on dentin, bringing about specific changes in its mechanical property leading to the brittleness of the tooth. When used at a higher concentration, 1g/ml, TAP treatment causes a significant reduction in microhardness at 500 µm from the pulp dentin complex compared with MTA at the same concentration. This is because of minocycline, which causes calcium chelation from the dentin [31].

Effect on Tooth Colour

One of the major drawbacks of TAP is discolouration of the tooth, for which minocycline is responsible. The problem can be resolved by the use of other medicaments like amoxicillin and cefaclor. The use of dentin bonding agents also, to a great extent, has been proven to prevent tooth discolouration [32].

Effect on Stem Cells

TAP is an important material used for the purpose of regeneration. It plays a significant role in preserving the health of the apical stem cell and, thus, in providing a microbe-free environment, enabling the stem cells to proliferate and help in regeneration [33]. Though materials like calcium hydroxide can be used, their toxic effect on the apical papilla leads to the avoidance of the use of such materials. In contrast, TAP has proven to overcome this drawback, proving to be the best intracanal disinfecting material with superior properties.

Limitations of TAP

Tooth discolouration is one of the major drawbacks of TAP. Studies have found that TAP was most linked to discolouration when compared to other antibiotic pastes like Ledermix, polyantibiotic paste, and Septomixine Forte. As a result, in some cases, the use of double antibiotic pate (DAP) containing only ciprofloxacin and metronidazole has been suggested. In other investigations, it was discovered that using DAP or TAP for a month reduced dentin microhardness considerably [33].

## Conclusions

For the success of the endodontic treatment, careful and complete eradication of pathogenic microbiota from the infected root canal space is very much required. Though mechanical preparation is the first step, it doesn't help in the complete disinfection of the space. Non-instrumentation techniques such as tooth repair and strategies for maintaining a state favourable for pulp regeneration and revascularization should be explored, especially in cases where local administration of drugs, particularly antibiotics, has been shown to be ineffective. Amongst all, TAP has been shown to be the most effective. TAP, a mix of three different antibiotics, provides effective action against different microorganisms and produces outstanding results. The combined action of three different antibiotics also helps reduce the chances of microbial resistance. As there are several advantages and disadvantages associated with the different antimicrobial agents available, the selection of the best is essential. TAP undoubtedly has proven to produce effective results.
